# Determination of the impact of rainfall on road accidents in Thailand

**DOI:** 10.1016/j.heliyon.2021.e06061

**Published:** 2021-02-12

**Authors:** Kamolrat Sangkharat, John E. Thornes, Porntip Wachiradilok, Francis D. Pope

**Affiliations:** a School of Geography, Earth and Environmental Sciences, UK; b Public Health England, CRCE, Chilton, Oxford, UK; c National Institute for Emergency Medicine (NIEM), Thailand

**Keywords:** Ambulance service, Emergency service, Rainfall, Road accidents

## Abstract

The World Health Organization has highlighted that the number of deaths worldwide due to road accidents increases every year. It recommends that countries improve road safety for all people by providing sustainable and safe transport systems by 2030, efforts are especially required within Low Middle-Income Countries (LMICS). This study is the first to investigate the impact of rainfall on road accidents in Thailand. Thai emergency data were collected from the National Institute for Emergency Medicine (NIEM) between 2012 to 2018. A time-series design with generalized linear model (GLM) was applied to analyse the associations between road accidents and rainfall. The results are reported using relative risk (RR) at 95% confidence intervals compared with dry days. The effects of long-term trends, seasonality, day of the weeks, public holidays and other meteorological factors were controlled in the GLM. A meta-analysis was applied to summarise the estimate effect of rain groups stratified by the Northern and Southern provinces.

Findings reported a significant increase in road accidents due to high rainfall levels both in the Southern and the Northern provinces. The pooled estimate risks in the Southern provinces have higher estimated risks than the Northern provinces. Both Northern and Southern provinces showed the rain group with 10–20 mm/day having the highest pooled estimated risk with RR = 1.052, (95% CI: 1.026–1.079) and RR = 1.062, (95% CI: 1.043–1.082), respectively, while surprisingly, heavy rain with more than 20 mm/day reported a reduction of risks. Road accidents can therefore be associated with rainfall. It is recommended that rainfall is factored into ambulance forecast models and warning systems, allowing for improvements in ambulance service efficiency. Policymakers need to integrate road safety policies that reduce road accidents in wet weather.

## 1 Introduction

The World Health Organization (WHO, 2018) has highlighted that the number of deaths worldwide due to road accidents increases every year. Worldwide in 2016, 1.35 million people died as a result of a road accident. Children and young people, defined as those aged between 5 and 29 years, are the group most at risk with the highest number of road accidents. The WHO suggested countries should set a plan to reduce the number of road accidents, with a corresponding Sustainable Development Goal (SDG) target which aims to reduce the number of global deaths and injuries from road traffic accidents by 50% by 2020 and provide a sustainable and safe transport system by 2030. The WHO reported that the number of accidents and deaths from road accidents in low income countries did not decrease during 2013–2016 period, whereas declines in 48 middle and high income countries were observed.

Rain can affect driving in various different ways. From an optical perspective, rain can lessen the performance of headlamps, tail lights, braking lights and other indicator lights, it can also reduce the capacity of the driver to see clearly and it reduces the contrast between objects and the road making driving more difficult. From a mechanical perspective, wet roads can reduce the grip of the vehicles tires to the road, thereby making the driving conditions more dangerous.

Previous studies have reported significant positive associations between rainfall and road accidents (Bergel-Hayat et al., 2013; Brodsky and Hakkert, 1988; Hermans et al., 2006). Heavy rain is a danger because of the reduced road skid resistance associated with a wet road (Bernardin et al., 2014). However, some publications claim that rainfall can also be a protective factor with decreases in road accidents observed during heavy rain. A possible explanation for these observations could be a reduction of traffic during heavy rain (Bergel-Hayat et al., 2013; Yannis and Karlaftis, 2010), or slower driving to reduce risk (Brodsky and Hakkert, 1988). Another study investigated whether delay effects were prevalent, with a decrease in estimated risks effect found on the day following the rain event (Brijs et al., 2008).

Moreover, the role of rain upon road accidents can also be associated with other factors such as the type of road e.g. motorway or urban road (Bergel-Hayat et al., 2013). Previously, the pattern of association between meteorological variables and road accidents was investigated for short-term effects using linear regression (Keay and Simmonds, 2006). This study investigates the possible relationship between rainfall and ambulance dispatches via increases in road accidents.

Thailand is a middle-income country located in South East Asia. Records indicate that ambulance dispatches due to road accidents represent the highest volume of ambulance dispatches countrywide and in every province. Ambulance dispatches due to road accidents have increased each year and by 32.8% between 2012 to 2018 (ITEMS, 2018). Prior to this study, no studies in Thailand have investigated the impact of rainfall on ambulance dispatches due to road accidents. Furthermore, no study has examined the difference between the provinces located in the Northern and the Southern Thailand. The new understanding gained from this study can be translated to policymakers to help them improve road safety and risk management.

## 2 Method

### 2.1 Data

Thai ambulance dispatch data, via the Thai emergency number 1699 was acquired from the National Institute for Emergency Medicine (NIEM). The data records the number of ambulance dispatches resulting from road traffic accidents that led to a patient being taken to hospitals. The ambulance data was available for 76 provinces, which represents all provinces other than the Bangkok metropolitan administration, which has their data collated by the Erawan Medical Centre Bangkok. The data for the provinces in this study, includes the nine that form the Northern provinces and the 14 that form the Southern provinces in Thailand. Meteorological data (temperature, relative humidity and precipitation) were obtained from monitoring stations in each province and were provided by the Thai Meteorological Department (www.tmd.go.th) as daily averages. If there are two monitoring stations in a province, the station located in an urban area was chosen rather than a rural area. Daily meteorological and road traffic accident data were obtained between January 2012 and December 2018.

### 2.2 Statistics

Two statistical analyses were conducted in this study. Firstly, the effects of precipitation on road accidents were investigated and stratified by groups of different rain intensities, as measured by the daily rate of precipitation. The generalised linear time series model (GLM) is used routinely for investigating associations between health outcomes and exposure to environmental factors. The association was described as having a linear trend between rain and road accident (Bergel-Hayat et al., 2013). Where Y is the health outcome, which in this study is a road accident. The model was adjusted for confounding factors such as temperature (Temp), relative humidity (RH), day of week (DOW) and public holiday (H) and the natural cubic spline (ns) is used as a smoothing function for time with a degree of freedom (df) of 7 following Xu et al. (2017). Results were reported in relative risks (RR) with 95% confidence intervals (CIs) for an increase of rainfall per day compared with dry days and a delay effect for one day by using a distributed lag non-linear model (DLNM) package in R. β, γ, δ and ε are described in a vector of coefficients for DOW, Holiday, RH and Rain, respectively. The core model is similar to that developed in a previous study (Sangkharat et al., 2020) as shown in Eq. (1), which is used to describe the association of exposures to rainfall upon road accidents. The estimated effect of rainfall is calculated for the association occurring at lag 0 (rainfall and accident occur on the same day) and lag 1 (the accident occurring the day after the rain event).

(1) Log [E(Y)] = α + cb (Rain_group, lag = 0,1)+ ns (Time, df = 7∗7)+ β DOW + γHoliday + δRH+ εTemp

Rain groups are categorical variables (range: 0–6, 0 for no rain (rain = 0), 1 for rain groups 0 < rain<1 mm/day, 2 for rain group 1 ≤ rain<2 mm/day, 3 for rain group 2 ≤ rain<5 mm/day, 4 for rain group 5 ≤ rain<10 mm/day, 5 for rain group 10 ≤ rain<20 mm/day and 6 for rain group more than 20 mm/day (rain≥20) per day). Akaike's Information Criterion (AIC) was used to consider the number of degrees of freedom to be used in the analysis (Guo et al., 2018), with the lowest AIC chosen. The rainfall effect was estimated by the RR of each rain group compared with no rain days (rain groups = 0).

Secondly, a meta-analysis to pool the risk effects was conducted to report the association of an increase in rainfall per day across the region by rain groups. Meta-analysis with random effect was used to analyse the association between rainfall and ambulance dispatches caused by road accidents in the Northern and the Southern provinces. The heterogeneity was evaluated by Cochran Q test and *I*^*2*^ number. The *I*^*2*^ statistic (Higgins's *I*^*2*^ test statistic) provides an explanation of the dispersion of effect sizes in the meta-analysis, which identifies the heterogeneity between geographical regions. *I*^*2*^ = 0–30%, ≥30–50% and ≥50% were defined as low, moderate and high heterogeneity respectively. The p-value from Cochran's Q test reports a significant level at 0.10 (10%).

## 3 Results

### *3.1* Summary of statistics for meteorology (temperature, relative humidity and rainfall)

Daily meteorological variables are summarized in Table 1, Table S1, and Table S2. Mean precipitation is higher in the Southern provinces ranging from 4.3 ± 12.5 to 11.9 ± 24.7 mm/day compared to the Northern provinces 3.0 ± 8.4 to 5.1 ± 12.7 mm/day (Table 1). The daily averages observed during the study period show that the Northern provinces have lower average temperatures and relative humidity compared to the Southern provinces. The mean (±1SD) temperature in the Northern provinces ranged from 25.3 ± 3.1°C to 28.1 ± 2.5°C (Northern provinces) and from 27.0 ± 1.2°C to 28.8 ± 2.7°C (Southern provinces) (Table S1). The mean relative humidity in the Northern provinces ranged from 70.1 ± 10.6% to 77.9 ± 9.5%, while the mean in the Southern ranged from 77.8 ± 5.7% to 83.8 ± 5.7% (Table S2).

**Table 1:** Summary daily statistics for precipitation in Thailand during 2012–2018 over Northern and Southern provinces. All values in mm/day.

Province	Mean ± SD	Min	Percentiles			Max
			P25	P50	P75	
**Northern provinces**						
Chiang Rai	5.1 ± 12.7	0.0	0.0	2.0	2.9	147.1
Chiang Mai	3.0 ± 8.4	0.0	0.0	0.0	0.9	124.8
Nan	3.2 ± 8.5	0.0	0.0	0.0	0.8	136.0
Payao	3.2 ± 9.7	0.0	0.0	0.0	0.8	145.6
Phrae	3.2 ± 9.3	0.0	0.0	0.0	0.9	115.5
Mae Hong Son	3.3 ± 8.4	0.0	0.0	0.0	3.3	74.8
Lampang	3.2 ± 9.3	0.0	0.0	0.0	1.2	115.9
Lamphun	3.1 ± 9.3	0.0	0.0	0.2	5.0	92.2
Uttaradit	3.6 ± 11.1	0.0	0.0	0.4	5.6	128.6

**Southern provinces**						
Krabi	6.5 ± 16.1	0.0	0.0	0.0	4.8	159.0
Chumphon	5.5 ± 14.9	0.0	0.0	0.0	3.6	250.3
Trang	6.7 ± 14.5	0.0	0.0	0.0	6.5	149.0
Nakhon Si Thammarat	7.7 ± 24.6	0.0	0.0	0.0	4.5	405.0
Narathiwat	8.2 ± 22.6	0.0	0.0	0.0	5.1	441.6
Pattani	5.3 ± 14.8	0.0	0.0	0.0	3.1	219
Phang Nga	11.9 ± 24.7	0.0	0.0	0.6	12.8	288.8
Phatthalung	6.4 ± 18.3	0.0	0.0	0.0	4.0	302.4
Phuket	7.1 ± 15.5	0.0	0.0	0.0	7.0	177.0
Ranong	12.9 ± 25.5	0.0	0.0	0.5	14.0	208.0
Satun	6.6 ± 14.7	0.0	0.0	0.0	6.4	192.6
Songkhla	6.6 ± 19.2	0.0	0.0	0.0	4.2	290.5
Surat Thani	4.3 ± 12.5	0.0	0.0	0.0	3.0	274.1
Yala	6.4 ± 16.7	0.0	0.0	0.0	4.4	197.3

Abbreviations SD: standard deviation, Px xth: percentile, Min: minimum, Max: maximum.

### 3.2 The association between rainfall and ambulance dispatches due to road accidents

The frequency of road accidents were compared between the different provinces. Most provinces show a consistent pattern, with a higher number of accidents found during the months of October to December. Investigation for day of the week effects, revealed a smaller number of road accidents at the weekend compared to weekdays as shown in Supplementary data.

The amount of road accidents associated with ambulance dispatches varied by the quantity of rain. For dry days, the daily average number of road accidents ranged from 3.5 calls in Mae Hong Son to 39.4 calls in Chiang Mai in the Northern provinces. In the Southern provinces, the number ranged from 4.6 calls in Pattani to 24.3 calls in Songkhla. For the 0–1 mm/day group, the average was from 2.9 to 35.2 in the Northern provinces, while there was 3.4–23.7 in the Southern provinces. For 1 ≤ rain<2 mm group, average road accidents were reported for 3.2 to 36.2 in the Northern provinces and 3.7 to 24.6 in the Southern provinces (Table 2).

**Table 2:** Summary for road accident dispatches from 2012 to 2018 stratified by rainfall groups.

Province	Dry day		0 < rain<1 (mm/day)		1 ≤ rain<2 (mm/day)		2 ≤ rain<5 (mm/day)		5 ≤ rain<10 (mm/day)		10 ≤ rain<20 (mm/day)		rain≥20 (mm/day)	
	N	x̄ ± SD	N	x̄ ± SD	N	x̄ ± SD	N	x̄ ± SD	N	x̄ ± SD	N	x̄ ± SD	N	x̄ ± SD
**Northern provinces**														
Chiang Rai	1569	22.5 ± 7.7	167	19.0 ± 6.2	100	18.2 ± 6.5	183	19.1 ± 7.3	155	18.6 ± 6.9	161	18.8 ± 6.9	222	19.4 ± 7.1
Chiang Mai	1694	39.4 ± 9.9	229	35.2 ± 8.9	101	36.2 ± 8.9	147	36.4 ± 10.4	134	36.3 ± 10.2	125	34.8 ± 9.1	127	32.6 ± 7.4
Nan	1739	8.4 ± 4.1	199	7.0 ± 3.7	104	7.2 ± 3.6	147	7.5 ± 4.8	124	7.1 ± 3.5	116	6.8 ± 3.4	128	6.9 ± 3.3
Payao	1760	8.0 ± 4.0	169	7.0 ± 3.1	80	6.8 ± 3.7	165	7.1 ± 3.8	148	7.2 ± 3.7	110	7.4 ± 3.6	125	7.0 ± 3.7
Phrae	1695	5.0 ± 2.8	229	4.6 ± 2.7	87	4.8 ± 2.4	150	4.2 ± 2.4	138	4.5 ± 2.7	126	4.7 ± 2.8	132	4.5 ± 2.8
Mae Hong Son	1594	3.5 ± 2.4	200	2.9 ± 2.1	119	3.2 ± 2.3	175	2.8 ± 2.1	208	3.0 ± 2.4	141	2.8 ± 2.1	118	2.8 ± 1.9
Lampang	1723	16.0 ± 8.0	174	15.7 ± 9.4	97	14.3 ± 6.1	169	14.9 ± 7.6	145	14.8 ± 8.5	126	15.4 ± 5.7	123	14.6 ± 6.6
Lamphun	1808	10.7 ± 4.4	183	9.7 ± 4.3	81	9.6 ± 4.6	128	10.0 ± 4.2	116	9.2 ± 3.9	115	10.3 ± 4.8	126	10.1 ± 4.1
Uttaradit	1786	8.5 ± 3.6	154	7.6 ± 3.3	101	8.2 ± 3.5	144	7.7 ± 3.5	123	7.7 ± 3.5	94	7.9 ± 2.9	155	7.7 ± 3.2

**Southern provinces**														
Krabi	1482	11.9 ± 6.1	141	12.4 ± 6.5	117	11.5 ± 7.3	182	11.7 ± 6.3	174	11.1 ± 5.4	182	11.3 ± 5.8	279	11.7 ± 5.6
Chumphon	1427	10.1 ± 4.4	244	9.8 ± 4.5	121	10.2 ± 4.0	196	9.8 ± 3.9	175	9.8 ± 4.0	165	10.2 ± 4.2	229	9.4 ± 5.0
Trang	1288	16.6 ± 7.7	206	16.2 ± 7.1	138	16.9 ± 9.1	221	15.5 ± 7.9	214	16.8 ± 12.6	212	15.3 ± 7.8	278	14.8 ± 7.3
Nakhon Si Thammarat	1390	19.8 ± 6.6	217	20.3 ± 6.9	127	20.5 ± 6.3	202	19.2 ± 6.2	186	18.7 ± 6.1	175	20.2 ± 7.0	259	18.5 ± 6.8
Narathiwat	1456	6.2 ± 3.0	174	6.2 ± 3.3	101	6.6 ± 3.9	180	5.7 ± 3.1	173	6.2 ± 3.3	170	6.2 ± 3.3	303	6.1 ± 3.3
Pattani	1552	4.6 ± 2.9	168	4.3 ± 2.9	105	4.5 ± 3.0	194	4.8 ± 2.8	155	4.7 ± 3.0	180	4.5 ± 3.0	203	4.8 ± 3.1
Phang Nga	1160	6.5 ± 3.5	165	6.3 ± 3.8	114	7.1 ± 4.1	206	6.6 ± 3.2	185	6.4 ± 3.5	239	5.8 ± 3.6	488	5.6 ± 3.0
Phatthalung	1424	7.7 ± 3.4	231	7.6 ± 4.2	108	7.4 ± 3.9	206	7.8 ± 3.6	174	7.4 ± 3.7	175	7.9 ± 4.0	239	6.7 ± 3.3
Phuket	1298	10.4 ± 7.0	205	9.9 ± 6.8	115	9.8 ± 6.7	204	10.0 ± 8.3	211	9.6 ± 6.8	221	10.7 ± 7.0	303	9.1 ± 6.8
Ranong	1141	3.6 ± 2.4	202	3.4 ± 2.3	91	3.7 ± 2.3	184	3.4 ± 2.5	194	3.2 ± 2.4	213	2.9 ± 2.1	532	2.9 ± 1.9
Satun	1308	6.1 ± 3.5	206	5.7 ± 3.4	122	5.3 ± 2.7	223	5.5 ± 3.3	204	5.3 ± 2.8	212	5.5 ± 3.4	282	5.6 ± 3.1
Songkhla	1439	24.0 ± 9.4	190	24.2 ± 9.4	118	24.6 ± 9.5	200	24.2 ± 9.6	196	23.2 ± 9.4	175	23.8 ± 9.5	239	23.4 ± 9.8
Surat Thani	1459	24.3 ± 7.1	234	23.7 ± 7.0	131	23.1 ± 6.4	229	23.7 ± 7.1	188	23.0 ± 7.2	165	24.1 ± 7.7	151	22.7 ± 6.9
Yala	1397	7.5 ± 3.3	213	7.2 ± 3.4	111	7.5 ± 3.7	216	7.1 ± 3.3	170	7.6 ± 3.6	192	7.0 ± 3.5	239	6.8 ± 3.2

To investigate the effect of rain on road accidents, the following variables were removed from the data: seasonality, public holiday, day of week, long-term trend and the temperature and relative humidity weather variables. Results indicate that the amount of rain is significantly associated with road accidents in most provinces (Table 3). Lags of 0 generated the most significant results. In Northern Thailand, five provinces had estimated results that were significant at lag 0 for the estimated effects compared to dry rain days. The largest single estimated effect was observed when the rain amount was 1 ≤ rain <2 mm/day at lag 0 in Phrae province (RR = 1.159, 95% CI: 1.027–1.308). For three provinces: Chiang Mai, Phrae and Lamphun, the most significant results occurred when the rain was in the range 10 ≤ rain <20 mm, where RR = 1.050 (95% CI: 1.004–1.067), RR = 1.127 (95% CI: 1.011–1.256) and RR = 1.111 (95% CI: 1.022–1.208), respectively. For rain in the ranges 5 ≤ rain <10 mm and greater than 20 mm were associated with road accidents in two provinces. Chaing Mai and Phrae estimated results were higher in road accidents at rain 5 ≤ rain <10 mm with RR = 1.091 (95% CI: 1.046–1.139) and RR = 1.115 (95% CI: 1.002–1.240) respectively. Chaing Rai and Lamphun observed a significant association at high rain (rain ≥20 mm), RR = 1.080 (95% CI: 1.028–1.135) and RR = 1.098 (95% CI: 1.011–1.192), respectively. Four provinces including Nan, Payao, Mae Hong Son and Uttaradit did not find any significant association. In addition, there were no significant associations between rain effects and road accidents at lag 1.

**Table 3:** The estimated effect of the association between rainfall and road accidents with 95 % CI shown in brackets by various lags.

Province	0<rain<1 mm/day		1≤rain<2 mm/day		2≤rain<5 mm/day		5≤rain<10 mm/day		10≤rain<20 mm/day		rain ≥20 mm/day	
	Lag 0	Lag 1	Lag 0	Lag 1	Lag 0	Lag 1	Lag 0	Lag 1	Lag 0	Lag 1	Lag 0	Lag 1
**Northern Provinces**												
Chiang Rai	0.960 (0.914–1.008)	0.931 (0.887–0.978)	0.982 (0.922–1.046)	0.951 (0.894–1.013)	1.020 (0.972–1.071)	0.956 (0.910–1.004)	1.000 (0.947–1.055)	0.972 (0.921–1.025)	1.035 (0.981–1.092)	0.930 (0.881–0.981)	**1.080 (1.028–1.135)**	0.929 (0.883–0.978)
Chiang Mai	1.015 (0.982–1.049)	1.000 (0.968–1.034)	1.032 (0.985–1.080)	0.961 (0.917–1.007)	**1.075 (1.032–1.119)**	1.010 (0.969–1.052)	**1.091 (1.046–1.139)**	1.009 (0.966–1.053)	**1.050 (1.004–1.097)**	1.005 (0.961–1.051)	0.986 (0.941–1.033)	0.949 (0.906–0.994)
Nan	1.000 (0.927–1.078)	1.022 (0.949–1.101)	1.038 (0.939–1.148)	0.959 (0.865–1.063)	1.072 (0.984–1.169)	1.037 (0.951–1.131)	1.058 (0.962–1.163)	1.030 (0.937–1.133)	1.036 (0.936–1.147)	0.969 (0.874–1.075)	1.039 (0.941–1.146)	0.995 (0.901–1.099)
Payao	0.962 (0.885–1.046)	0.951 (0.874–1.035)	0.931 (0.827–1.047)	1.016 (0.907–1.138)	1.032 (0.948–1.124)	1.011 (0.928–1.101)	1.019 (0.929–1.117)	1.033 (0.943–1.132)	1.035 (0.937–1.144)	1.005 (0.908–1.112)	1.002 (0.906–1.108)	1.095 (0.993–1.207)
Phrae	1.086 (0.999–1.180)	1.054 (0.970–1.146)	**1.159 (1.027–1.308)**	1.056 (0.932–1.197)	1.027 (0.926–1.139)	1.035 (0.934–1.147)	**1.115 (1.002–1.240)**	1.083 (0.974–1.206)	**1.127 (1.011–1.256)**	1.095 (0.982–1.222)	1.105 (0.990–1.233)	0.943 (0.840–1.058)
Mae Hong Son	0.988 (0.880–1.109)	0.953 (0.847–1.078)	1.109 (0.966–1.274)	1.021 (0.882–1.182)	1.016 (0.894–1.154)	1.025 (0.902–1.166)	1.086 (0.962–1.227)	1.087 (0.962– 1.229)	1.023 (0.888–1.179)	1.104 (0.960–1.270)	1.013 (0.870–1.179)	1.029 (0.884–1.199)
Lampang	**1.086 (1.003–1.176)**	0.975 (0.900–1.058)	1.022 (0.918–1.138)	0.974 (0.875–1.084)	1.053 (0.966–1.147)	0.985 (0.903–1.073)	1.039 (0.948–1.138)	0.959 (0.875–1.052)	1.062 (0.963–1.172)	0.935 (0.845–1.034)	1.040 (0.940–1.151)	0.933 (0.842–1.035)
Lamphun	0.990 (0.926–1.058)	1.048 (0.982–1.118)	0.990 (0.900–1.089)	0.983 (0.895–1.080)	1.042 (0.963–1.127)	0.957 (0.884–1.037)	1.010 (0.927–1.101)	0.951 (0.872–1.037)	**1.111 (1.022–1.208)**	0.966 (0.886–1.053)	**1.098 (1.011–1.192)**	0.952 (0.875–1.037)
Uttaradit	1.009 (0.937–1.086)	1.004 (0.932–1.080)	1.045 (0.959–1.139)	0.976 (0.894–1.066)	0.982 (0.909–1.060)	0.968 (0.896–1.045)	1.000 (0.919–1.088)	0.950 (0.872–1.034)	1.023 (0.929–1.125)	0.968 (0.878–1.066)	1.010 (0.934–1.093)	1.022 (0.945–1.105)
**Southern Provinces**												
Krabi	1.056 (0.986–1.130)	1.019 (0.951–1.092)	1.077 (0.997–1.163)	0.991 (0.915–1.073)	**1.068 (1.002–1.138)**	0.993 (0.931–1.060)	1.058 (0.989–1.132)	1.007 (0.941–1.078)	1.034 (0.968–1.104)	0.977 (0.913–1.044)	**1.113 (1.050–1.179)**	0.987 (0.929–1.049)
Chumphon	1.015 (0.956–1.078)	0.993 (0.935–1.054)	1.084 (0.999–1.176)	0.948 (0.871–1.032)	1.036 (0.966–1.111)	0.982 (0.916–1.054)	1.055 (0.980–1.136)	0.942 (0.873–1.016)	**1.119 (1.036–1.208)**	1.018 (0.942–1.100)	1.044 (0.966–1.129)	1.026 (0.950–1.109)
Trang	1.027 (0.951–1.108)	0.989 (0.916–1.069)	1.068 (0.976–1.168)	1.026 (0.937–1.123)	1.004 (0.927–1.086)	0.973 (0.899–1.053)	**1.086 (1.003–1.176)**	1.006 (0.927–1.090)	1.044 (0.957–1.140)	0.935 (0.855–1.021)	1.023 (0.939–1.116)	0.918 (0.841–1.002)
Nakhon Si Thammarat	1.031 (0.988–1.075)	1.001 (0.959–1.044)	1.042 (0.988–1.100)	0.969 (0.918–1.024)	1.021 (0.975–1.069)	1.011 (0.965–1.058)	1.001 (0.953–1.052)	1.003 (0.955–1.053)	**1.053 (1.001–1.107)**	0.988 (0.939–1.040)	1.034 (0.981–1.090)	0.993 (0.941–1.046)
Narathiwat	1.028 (0.947–1.115)	1.024 (0.944–1.110)	1.097 (0.991–1.214)	1.030 (0.929–1.142)	0.970 (0.891–1.055)	1.016 (0.936–1.103)	1.020 (0.939–1.108)	0.987 (0.908–1.073)	1.056 (0.970–1.150)	0.984 (0.902–1.073)	1.076 (1.000–1.160)	0.996 (0.923–1.075)
Pattani	1.023 (0.932–1.123)	1.045 (0.954–1.146)	1.078 (0.962–1.209)	1.003 (0.893–1.126)	1.061 (0.973–1.157)	0.993 (0.910–1.084)	1.057 (0.957–1.167)	0.958 (0.866–1.061)	1.098 (0.996–1.210)	1.043 (0.946–1.149)	**1.157 (1.049–1.275)**	0.984 (0.889–1.090)
Phang Nga	1.039 (0.951–1.135)	1.029 (0.944–1.121)	**1.174 (1.064–1.297)**	1.017 (0.918–1.126)	**1.122 (1.034–1.218)**	0.998 (0.919–1.084)	**1.127 (1.032–1.230)**	0.969 (0.885–1.060)	1.054 (0.967–1.149)	0.926 (0.849–1.010)	**1.097 (1.013–1.187)**	0.897 (0.827–0.973)
Phatthalung	1.057 (0.993–1.124)	0.962 (0.903–1.025)	0.980 (0.897–1.070)	1.003 (0.920–1.094)	1.068 (0.997–1.143)	0.987 (0.921–1.058)	1.014 (0.940–1.093)	0.977 (0.906–1.054)	1.092 (1.013–1.177)	1.001 (0.927–1.081)	0.969 (0.896–1.048)	0.951 (0.879–1.029)
Phuket	1.038 (0.971–1.110)	0.966 (0.903–1.034)	1.011 (0.928–1.100)	0.982 (0.902–1.069)	1.066 (0.996–1.141)	0.992 (0.926–1.063)	1.070 (0.998–1.147)	0.961 (0.895–1.032)	**1.096 (1.026–1.171)**	0.917 (0.855–0.982)	**1.100 (1.026–1.180)**	0.934 (0.869–1.004)
Ranong	1.018 (0.916–1.132)	1.021 (0.919–1.135)	1.129 (0.979–1.303)	1.086 (0.939–1.255)	1.074 (0.958–1.205)	1.021 (0.909–1.148)	1.017 (0.901–1.149)	1.046 (0.926–1.181)	0.960 (0.846–1.089)	1.047 (0.925–1.184)	1.054 (0.938–1.185)	1.042 (0.926–1.172)
Satun	1.007 (0.925–1.096)	0.948 (0.870–1.034)	0.960 (0.861–1.071)	1.008 (0.907–1.121)	1.032 (0.947–1.125)	0.985 (0.903–1.075)	1.030 (0.937–1.132)	0.974 (0.885–1.072)	1.079 (0.982–1.185)	0.999 (0.908–1.099)	1.043 (0.949–1.147)	0.996 (0.905–1.095)
Songkhla	1.033 (0.991–1.077)	1.015 (0.974–1.059	**1.055 (1.002–1.110)**	0.968 (0.918–1.020)	1.035 (0.993–1.079)	1.008 (0.966–1.051)	1.015 (0.971–1.060)	0.989 (0.947–1.033)	**1.060 (1.012–1.110)**	0.986 (0.941–1.034)	**1.074 (1.026–1.124)**	0.991 (0.946–1.038)
Surat Thani	1.002 (0.964–1.042)	**1.053 (1.014–1.094)**	1.003 (0.954–1.055)	1.043 (0.993–1.096)	1.025 (0.984–1.068)	1.041 (0.999–1.084)	1.015 (0.969–1.062)	1.025 (0.979–1.073)	**1.056 (1.00–61.109)**	**1.061 (1.010–1.114)**	1.052 (0.999–1.108)	1.031 (0.978–1.086)
Yala	1.051 (0.984–1.122)	1.043 (0.977–1.113)	1.064 (0.977–1.159)	0.969 (0.888–1.058)	1.060 (0.990–1.135)	0.993 (0.926–1.064)	**1.136 (1.055–1.223)**	1.003 (0.929–1.082)	1.048 (0.972–1.130)	0.963 (0.892–1.039)	1.077 (0.998–1.161)	0.980 (0.908–1.058)

Bold = Statistically significant at 95% CI.

In the Southern provinces, the estimated effect of rainfall was significantly associated with road accidents in ten provinces. The highest estimated effect was reported in Phang Nga (RR = 1.174, 95% CI: 1.064–1.297) and the remaining were Krabi (RR = 1.113, 95% CI: 1.050–1.179), Chumphon (RR = 1.119, 95% CI: 1.036–1.208), Trang (RR = 1.086, 95% CI: 1.003–1.176), Nakhon Si Thammarat (RR = 1.053, 95% CI: 1.001–1.107), Pattani (RR = 1.157, 95%CI: 1.049–1.275), Phuket (RR = 1.100, 95% CI: 1.026–1.180), Songkhla (RR = 1.074, 95% CI: 1.026–1.124), Surat Thani (RR = 1.061, 95% CI: 1.010–1.114) and Yala (RR = 1.136, 95% CI: 1.055–1.223). However, there were no significant associations between rainfall and road accidents in four provinces: Narathiwat, Phatthalung, Ranong and Satun. estimated effect at lag 1 in Surat Thani (RR = 1.053, 95% CI: 1.014–1.3094), with no significant result at lag 0.

For the estimated effects of lag 0 and lag 1, all results did not show delayed effects except Surat Thani for which the result at lag 1 was higher than the results at lag 0. The estimated effect at lag 1 in Surat Thani was significant (RR = 1.053, 95% CI: 1.014–1.3094), whilst at lag 0 it was insignificant. There were different patterns of the impact of rainfall in different provinces. Certain provinces showed significant increases in road accidents due to a rising rain volume, for example, in Chiang Rai and Phuket. Other provinces showed initial increases in accidents with rainfall, then reduced numbers of accidents as rainfally volume increased further. This pattern is clearly observed in Chiang Mai, Nan and Payao. Some provinces showed an increase initially, then a decline, followed by a rise again such as Phrae, Mae Hong Son, Lampang, Lamphun, Uttaradit, Krabi, Chumphon, Trang, Nakhon Si Thammarat, Narathiwat, Pattani, Phang Nga, Phattalung, Ranong, Satun, Songkhla, Surat Thani and Yala as shown in Figure S2.

### 3.3 Meta-analyses for pooled estimated risks of the association between rainfall and road accidents

**Figure 1:**
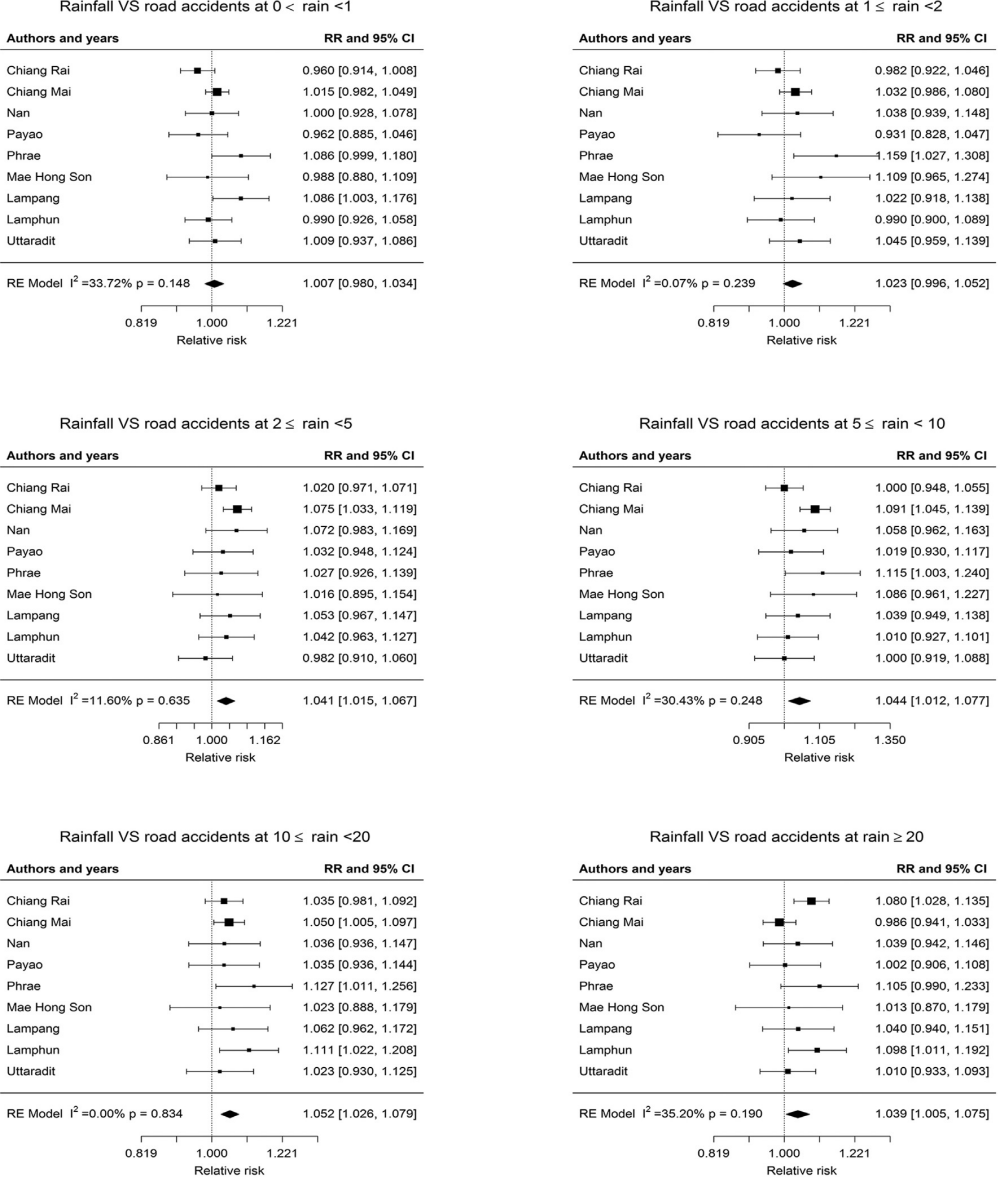
Forest plot for rainfall and road accidents at different rain volume (rain group) in the Northern provinces of Thailand.

**Figure 2:**
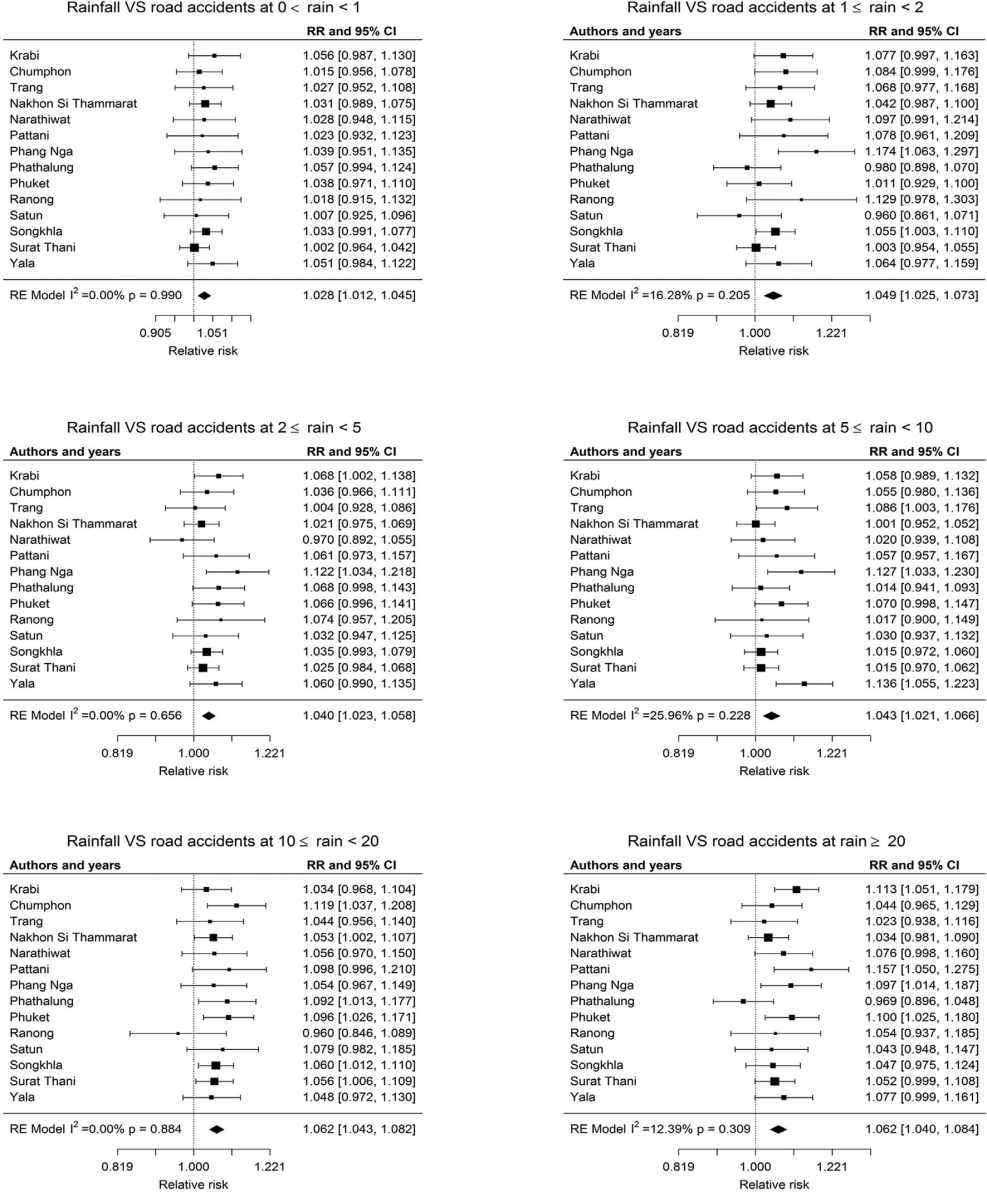
Forest plot for rainfall and road accidents at different rain volume (rain group) in the Southern provinces of Thailand.

The estimated effect from different provinces were pooled by regions and stratified by rain groups as shown in Figures 1 and 2. Rain groups are associated with an increase in road accidents in the Northern provinces for 2 ≤ rain<5 mm/day (RR = 1.041, 95%CI: 1.015–1.067), 5 ≤ rain<10 mm/day (RR = 1.044, 95%CI: 1.012–1.077), 10 ≤ rain<20 mm/day (RR = 1.052, 95%CI: 1.026–1.079) and greater than 20 mm/day (rain ≥20) (RR = 1.039, 95%CI: 1.005–1.075). However, no significant associations for rain group at 0 < rain<1 mm/day and 1 ≤ rain<2 mm/day were observed. Rain groups are associated with an increase in road accidents in the Southern provinces for all rain group stratifications: for 0 < rain<1 mm/day (RR = 1.028, 95%CI: 1.012–1.045), 1 ≤ rain<2 mm/day (RR = 1.049, 95% CI: 1.025–1.073), 2 ≤ rain<5 mm/day (RR = 1.040, 95% CI: 1.023–1.058), 5 ≤ rain<10 mm/day (RR = 1.043, 95% CI: 1.021–1.600), 10 ≤ rain<20 mm/day (RR = 1.062, 95% CI: 1.043–1.082) and greater than 20 mm/day (rain ≥20) (RR = 1.062, 95% CI: 1.040–1.084). Within the associated uncertainties, it is not possible to assign a statistically significant difference in association between road accident and rain volume between the Southern and Northern regions.

## 4 Discussion

The rainfall results reported a significant increase in ambulance dispatches due to road accidents in both the Southern and the Northern provinces. The observed results support similar evidence from a previous study (Bergel-Hayat et al., 2013). The pooled estimated RR for ambulance dispatches for road accidents due to rain in the Southern provinces was higher than the Northern provinces. The pooled estimate from nine provinces in the North had a significant association at rainfall level 2 ≤ rain <5 mm/day, 5 ≤ rain <10 mm/day, 10 ≤ rain <20 mm/day and rain > 20 mm/day, but not for lower rain at 0 < rain <1 mm/day and 1 ≤ rain <2 mm/day. The largest pooled estimated RR is 1.052 (95%CI: 1.026–1.079) at rainfall 10 ≤ rain <20 mm/day.

The result from the Northern provinces was different from the Southern findings, with an increase in road accident dispatches associated with all rain groups. The group of rain with 10–20 mm/day result had the highest pooled estimated risk (RR = 1.062, 95% CI: 1.043–1.082). These differences between areas can be explained in terms of geographic location (Guo et al., 2012), which is consistent with our findings that reported the amount of rainfall in the Southern province was higher than the Northern province because of Southwest monsoon.

Interestingly, our results reported that the association between road accident dispatches and rainfall did not have a linear relationship. The estimated effects for the heavy rain group (more than 20 mm/day) was lower than the rain volume 10–20 mm/day. These result are consistent with Bergel-Hayat et al. (2013) and Yannis and Karlaftis (2010) who reported the negative relationship between precipitation and accident on the road. The results are likely to be related to the behaviour of drivers due to the decline of traffic volume during heavy rain. In heavy rain, people are likely to change activity plans; and this behaviour is likely to be related to geographic areas. Previous studies mentioned that drivers change their behaviour on the days following rainfall resulting in a reduction of motorcycle risks (Brijs et al., 2008; Eisenberg, 2004).

Our findings can be advantageous for the public health surveillance systems. Policymakers should consider environmental factors when creating a strategy to improve the ambulance service and public health in general. Better prediction of the volume of ambulance calls due to changing environmental factors can improve the effectiveness of ambulance services. Raising people's awareness about the safety of driving under extreme weather conditions can help mitigate the associated risks. Adaptation techniques could include: checking the maintenance of vehicles regularly, driving carefully with reduced speed and using helmet if on a bike. WHO (2018) stressed that low and middle income countries need to take advantage of the experience of high income countries regarding road safety policies, road designs and good standard of driving behaviours.

This study has several limitations. Thai ambulance dispatch data did not contain demographic information data and geographical data disaggregated further than the province level. In addition, the road accident model could be further refined to investigate the association between rainfall and road accident by adding related factors such as the traffic volume, type of road, type of cars and speed of vehicle. Hence, further studies should take these variables into account to provide a more nuanced explanation of risk factors for the public health sector. Another study limitation is the use of daily data for ambulance and meteorological data. Going forward, it would likely be advantageous to investigate relationships between rain and ambulance dispatch data at an hourly time resolution.

## 5 Conclusion

This study provides clear evidence of the association between an increase in road accidents and rainfall. The results did not report a delay effect or the impact of lagged days. These results suggest a surveillance system for the association between environmental factors could be beneficially set up in Thailand. This can be a prevention measure to protect against environmental impacts. With this information, the Thai emergency services can improve the effectiveness of both staff and equipment. For this to be effective, health education about the impact of environmental factors is required. The outcomes from this study on Thai data is likely applicable to other countries at similar stages of development and meteorology.

## Declarations

### Author contribution statement

Kamolrat Sangkharat: Conceived and designed the experiments; Performed the experiments; Analyzed and interpreted the data;Wrote the paper.

John E. Thornes: Conceived and designed the experiments; Analyzed and interpreted the data; Wrote the paper.

Porntip Wachiradilok: Analyzed and interpreted the data; Contributed reagents, materials, analysis tools or data.

Francis D. Pope: Conceived and designed the experiments; Analyzed and interpreted the data; Contributed reagents, materials, analysis tools or data; Wrote the paper.

### Funding statement

This work was supported by Natural Environment Research Council and Medical Research Council (NE/P010997/1), Thai government PhD scholarship.

### Data availability statement

Data included in article.

### Declaration of interests statement

The authors declare no conflict of interest.

### Additional information

No additional information is available for this paper.

## Appendix A Supplementary data

Supplementary Data -V2.docx Supplementary Data -V2.docx
