# Supplementary material for: Determination of the impact of rainfall on road accidents in Thailand
Source: Heliyon. 2021 Feb 12;7(2):e06061. doi: 10.1016/j.heliyon.2021.e06061 (PMC7895724; doi:10.1016/j.heliyon.2021.e06061)
Supplement: Supplementary Data -V2.docx [file mmc1.docx]

# Supplementary Data

# Determination of the impact of Rainfall on Road Accidents in Thailand

**Figure S1** Seasonal (monthly) variation (right side) of total dispatches and day-of-week variation (left side) as shown in x-axis of ambulance dispatches caused by road accidents (calls) (y-axis) by using openair package in R software.

**Figure S2** Forest plot of the relative risk with 95% CI for road accidents with different rain group groups and different lagged days of province in the Northern and the Southern provinces. The DLNM package in the R software is allowed to study delay effects (lags) for providing the estimated effect between rainfall and road accidents via function cross-basis.

**Table S1** Summary daily statistics for temperature in Thailand during 2012-2018 over Northern and Southern provinces. All values in °C.

**Table S2** Summary daily statistics for relative humidity in Thailand during 2012-2018 over Northern and Southern provinces. All values in %.

| Monthly | Weekly |
| --- | --- |
| 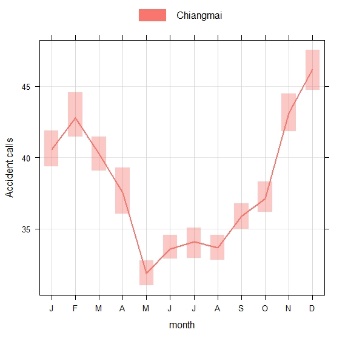 | 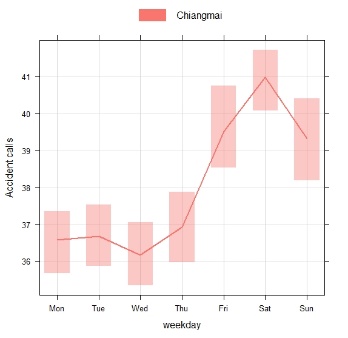 |
|  |  |
| 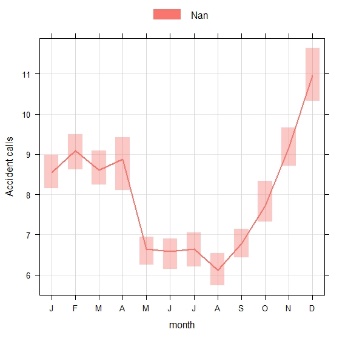 | 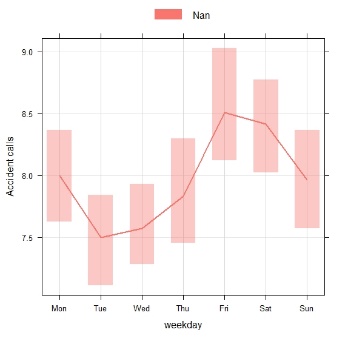 |
|  |  |
| 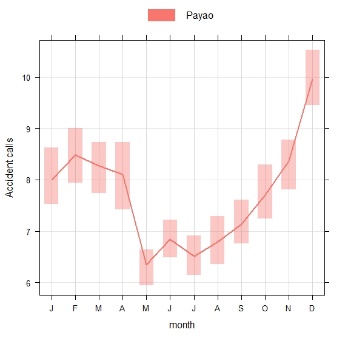 | 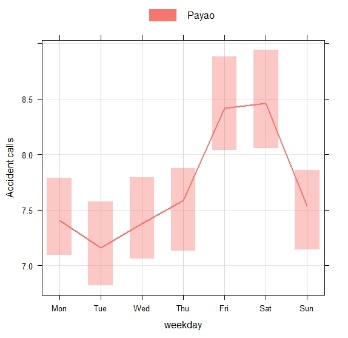 |
|  |  |
| 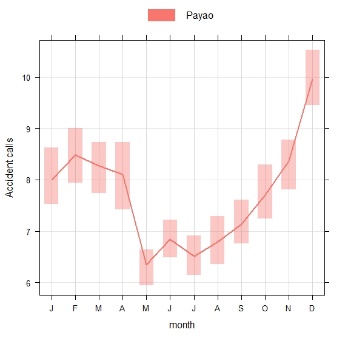 | 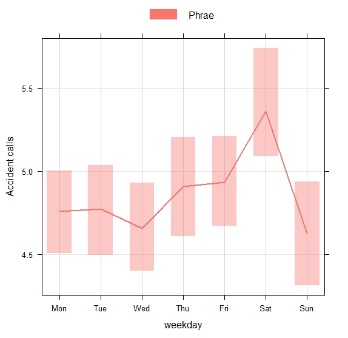 |
|  |  |
| 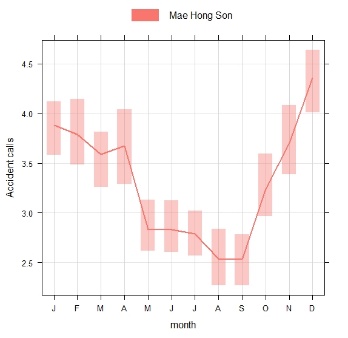 | 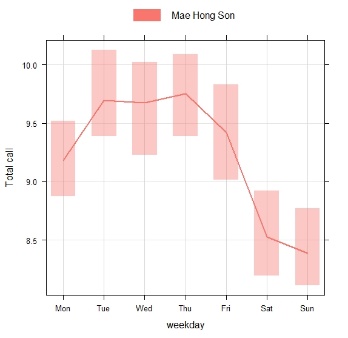 |
|  |  |
| 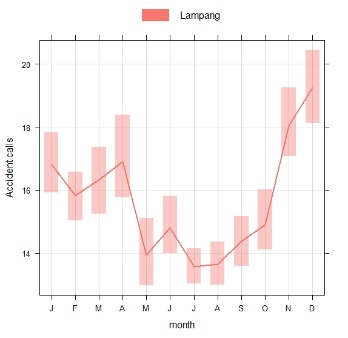 | 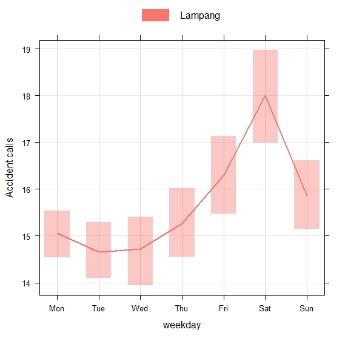 |
|  |  |
| 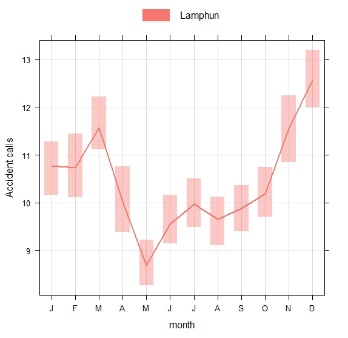 | 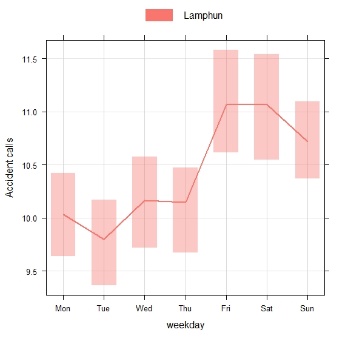 |
|  |  |
| 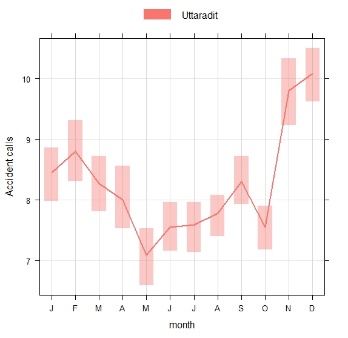 | 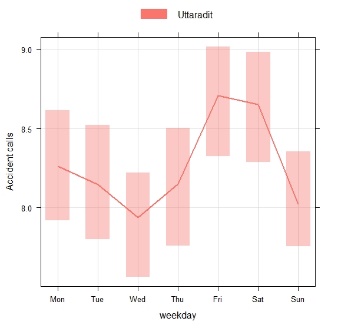 |
|  |  |
| 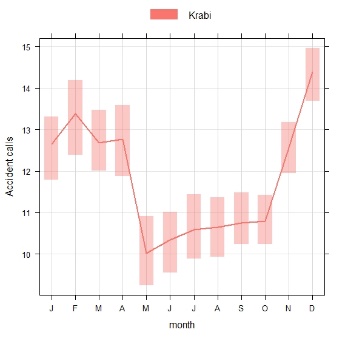 | 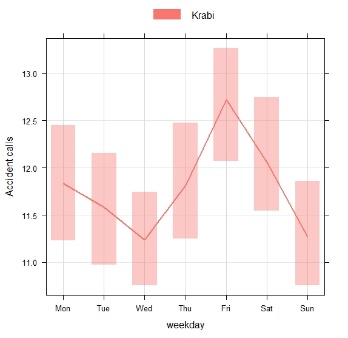 |
|  |  |
| 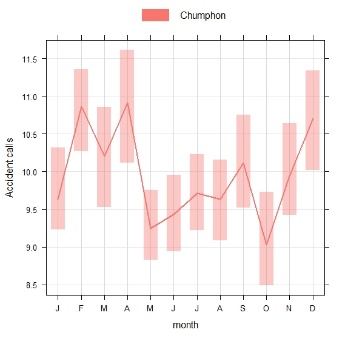 | 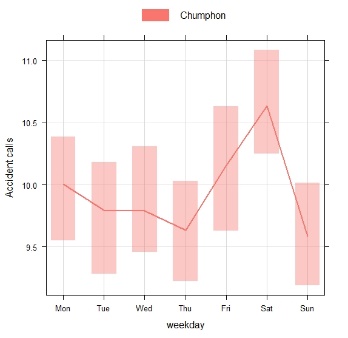 |
|  |  |
| 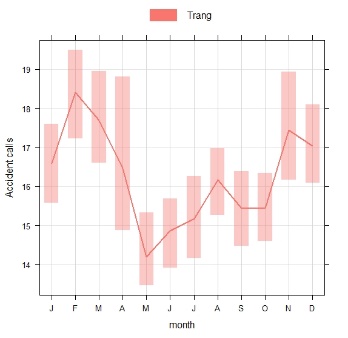 | 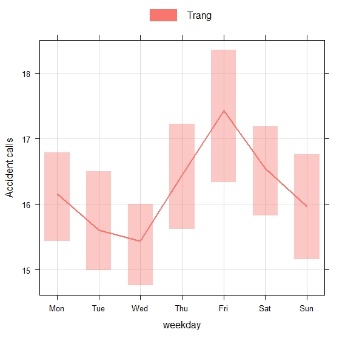 |
|  |  |
| 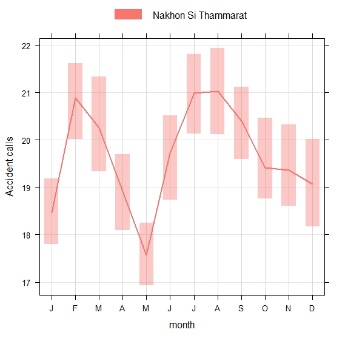 | 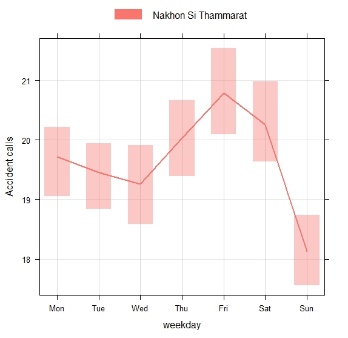 |
|  |  |
| 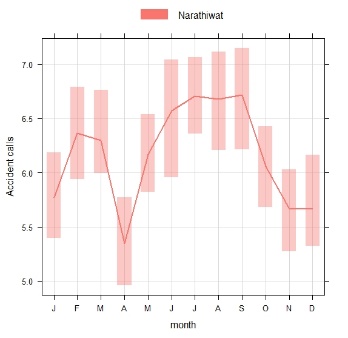 | 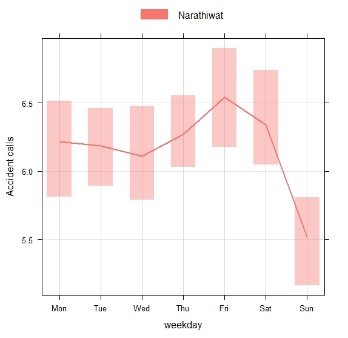 |
|  |  |
| 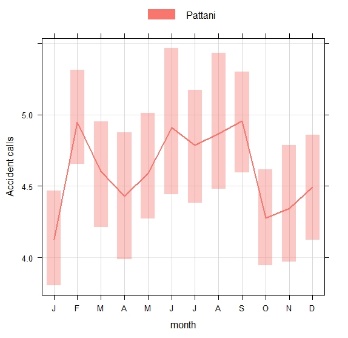 | 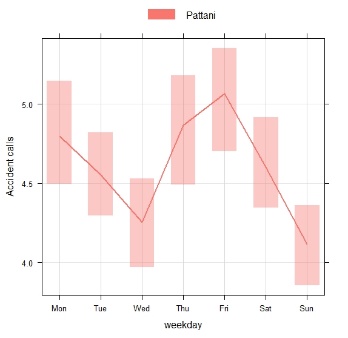 |
|  |  |
| 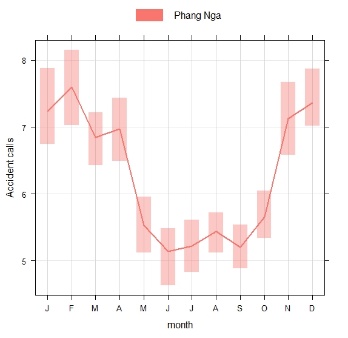 | 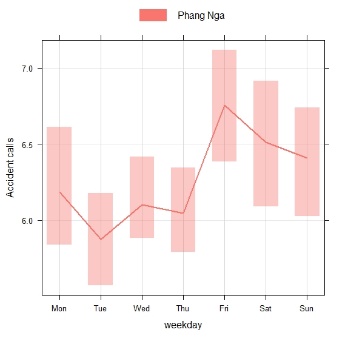 |
|  |  |
| 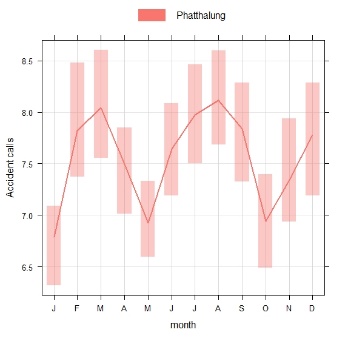 | 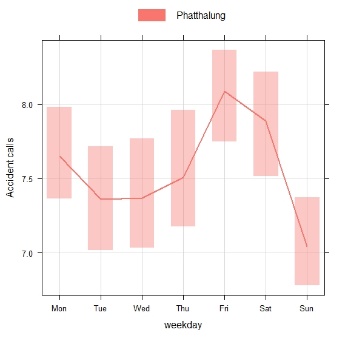 |
|  |  |
| 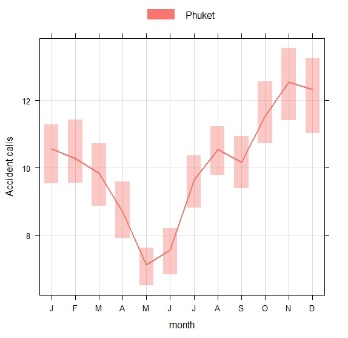 | 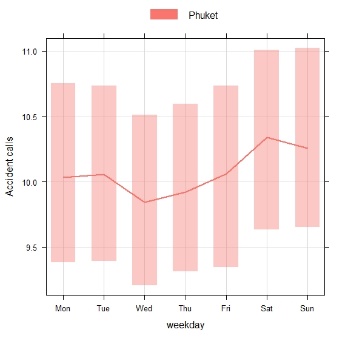 |
|  |  |
| 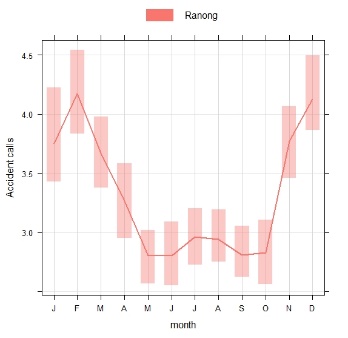 | 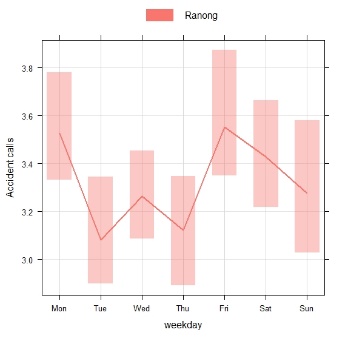 |
|  |  |
| 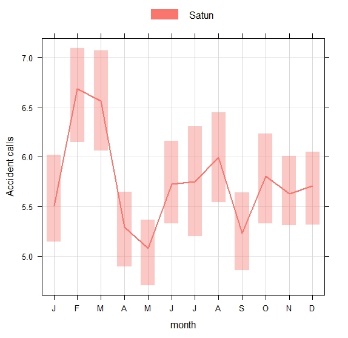 | 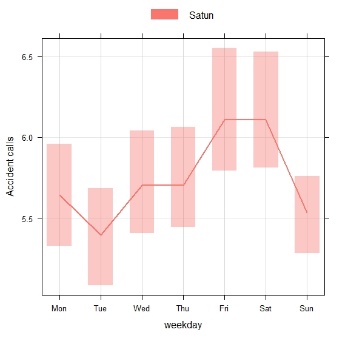 |
|  |  |
| 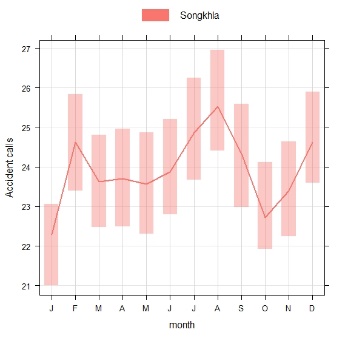 | 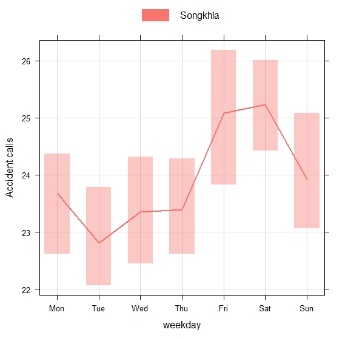 |
| 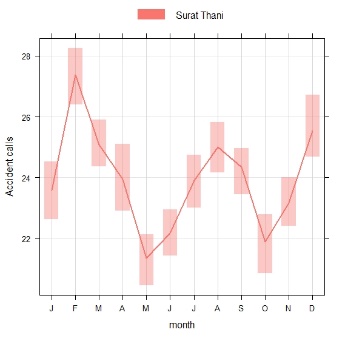 | 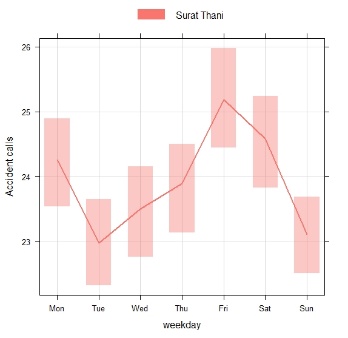 |
|  |  |
| 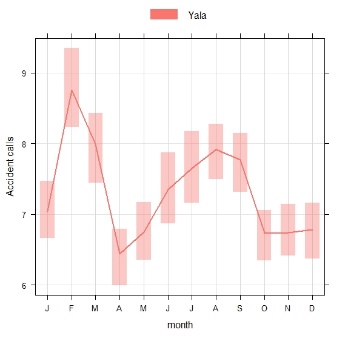 | 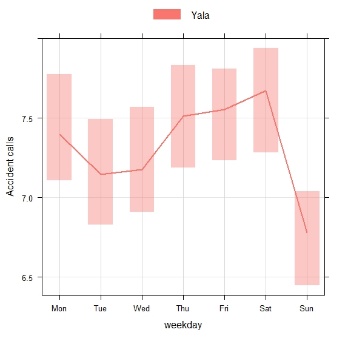 |

**Figure S1** Seasonal (monthly) variation (right side) of total dispatches and day-of-week variation (left side) as shown in x-axis of ambulance dispatches caused by road accidents (calls) (y-axis) by using openair package in R program.

| **Northern provinces** | |
| --- | --- |
| Chiang Rai | Chiang Mai |
| 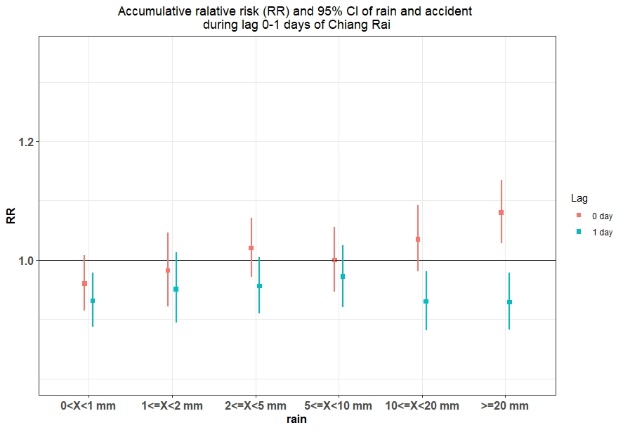 | 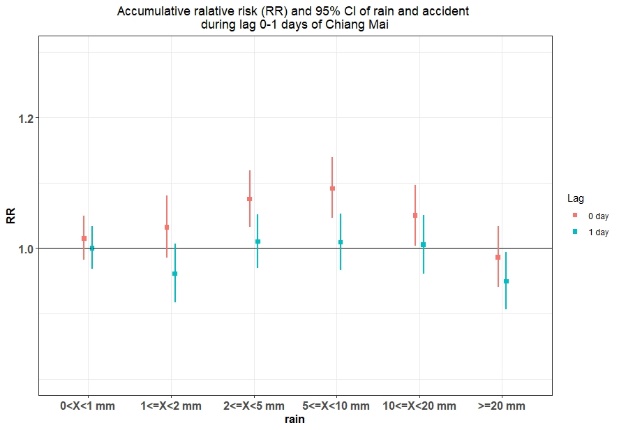 |
| Nan | Payao |
| 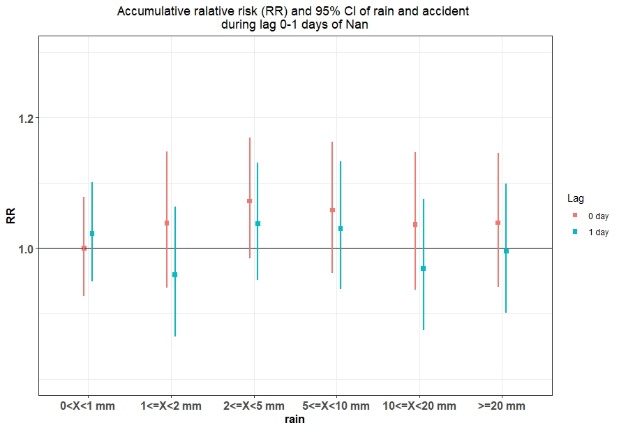 | 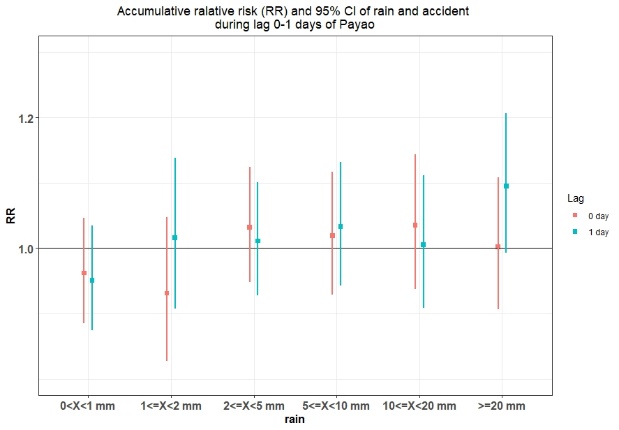 |
| Phrae | Mae Hong Son |
| 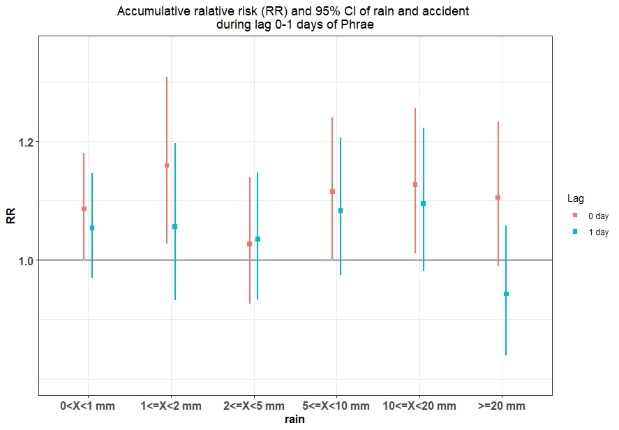 | 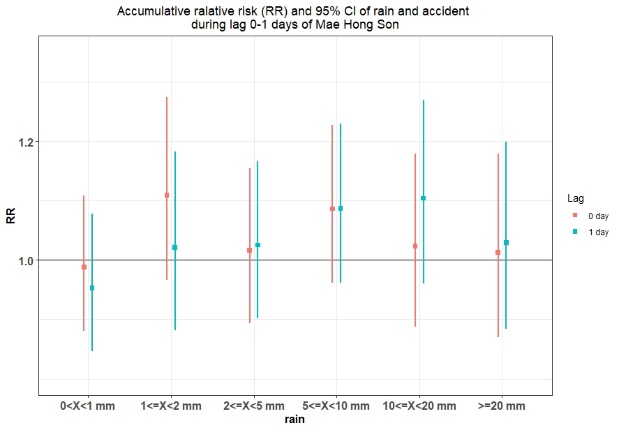 |
| Lampang | Lamphun |
| 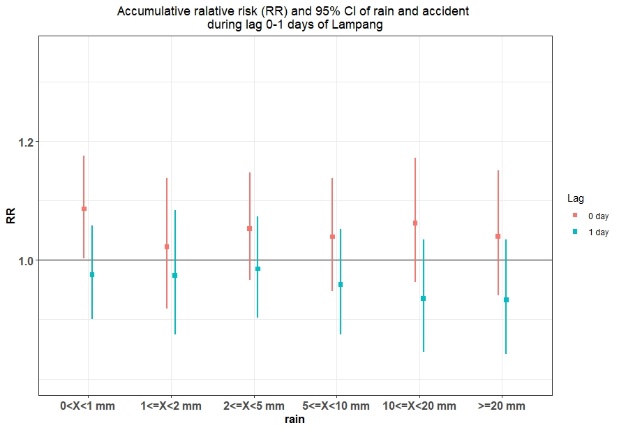 | 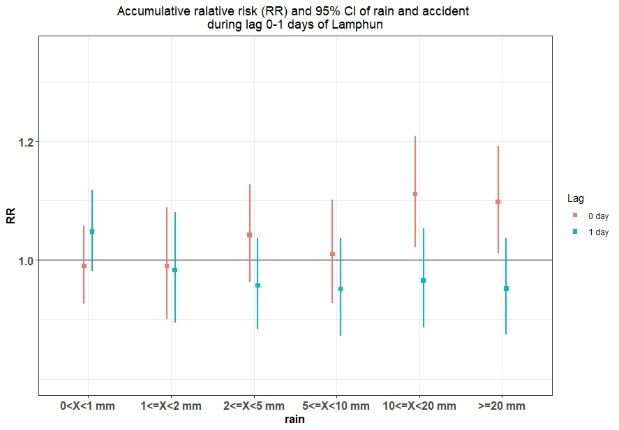 |
|  |  |
| Uttaradit |  |
| **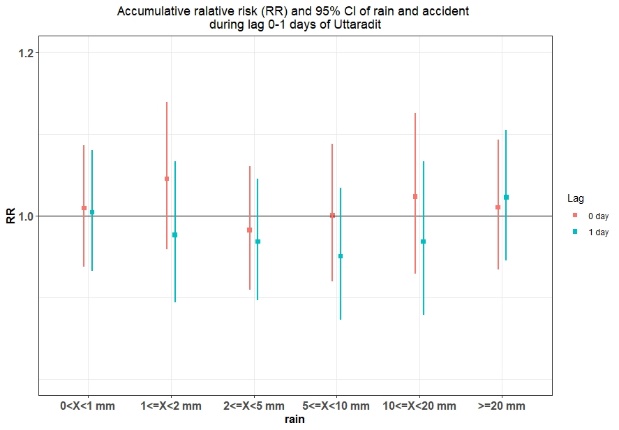** |  |
|  | |
| **Southern provinces** | |
| Krabi | Chumphon |
| 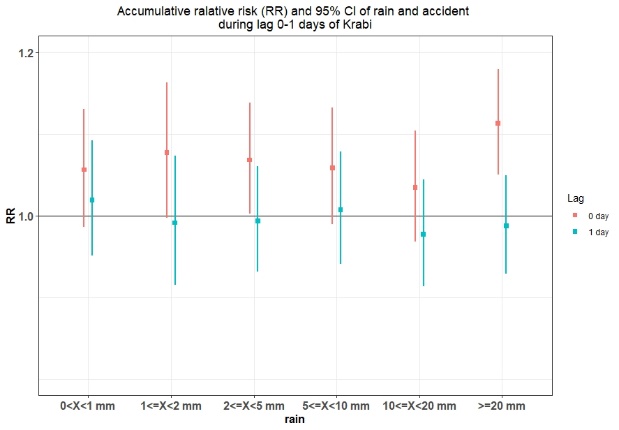 | 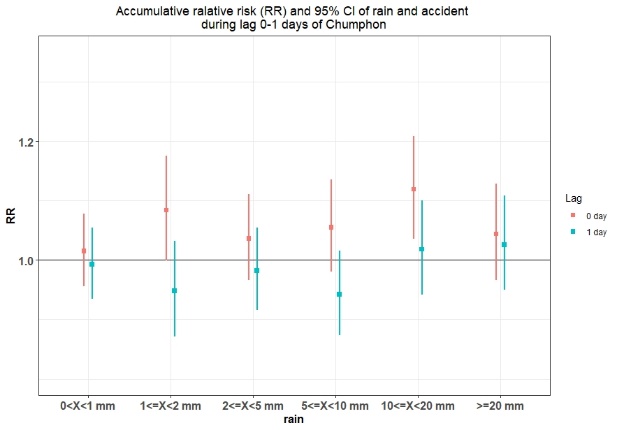 |
| Trang | Nakhon Si Thammarat |
| 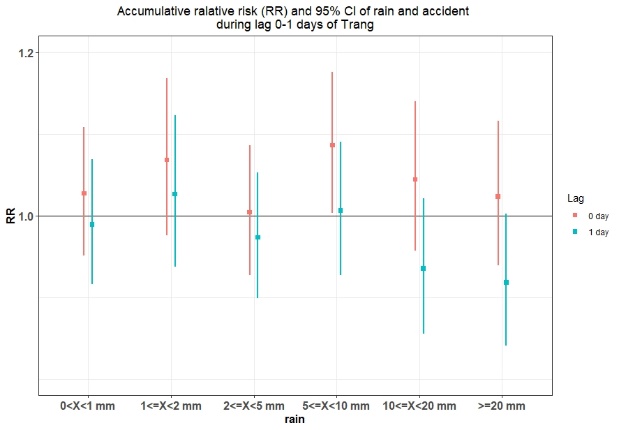 | 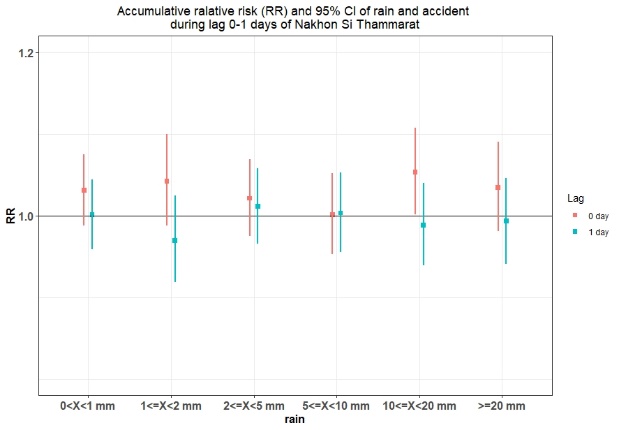 |
| Narathiwat | Pattani |
| 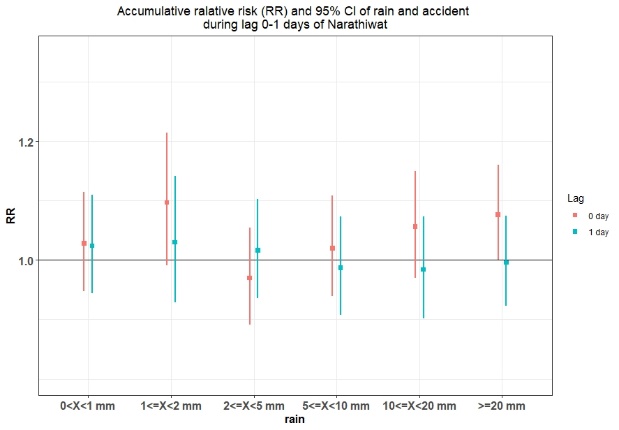 | 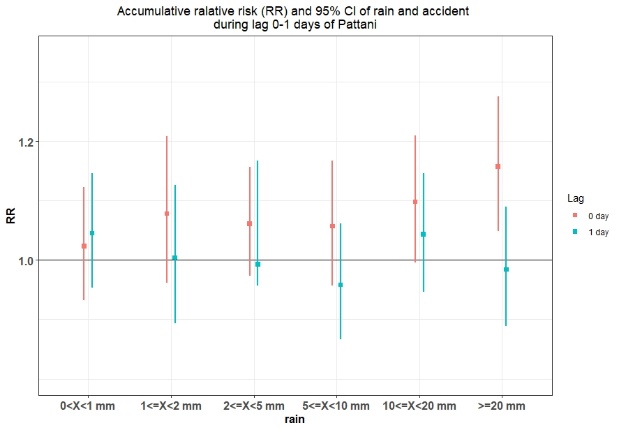 |
| Phang Nga | Phatthalung |
| 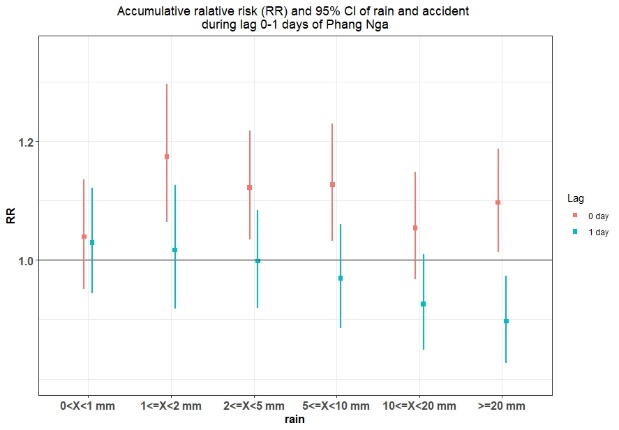 | 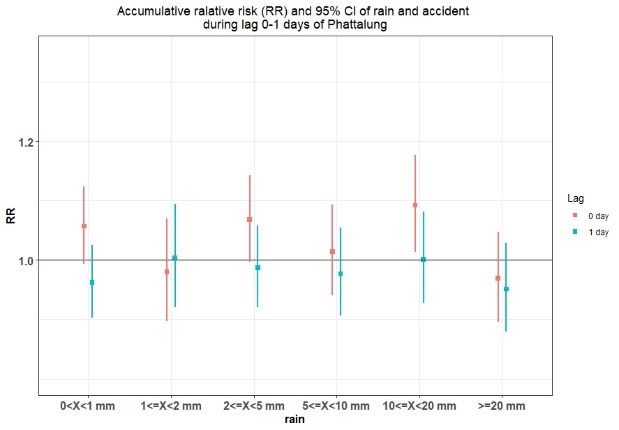 |
| Phuket | Ranong |
| 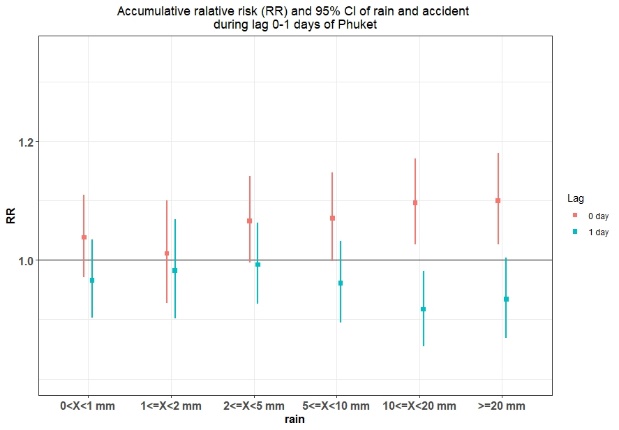 | 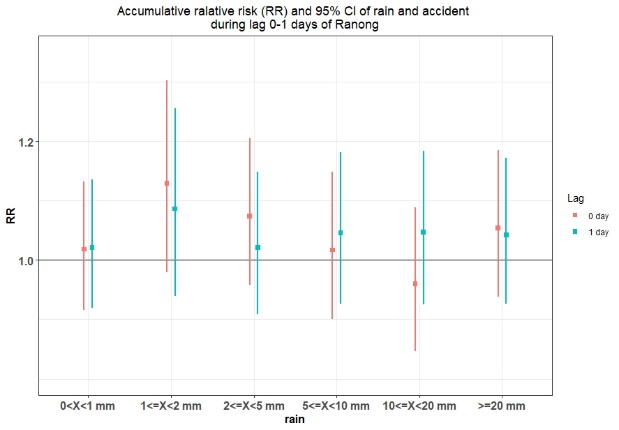 |
| Satun | Songkhla |
| 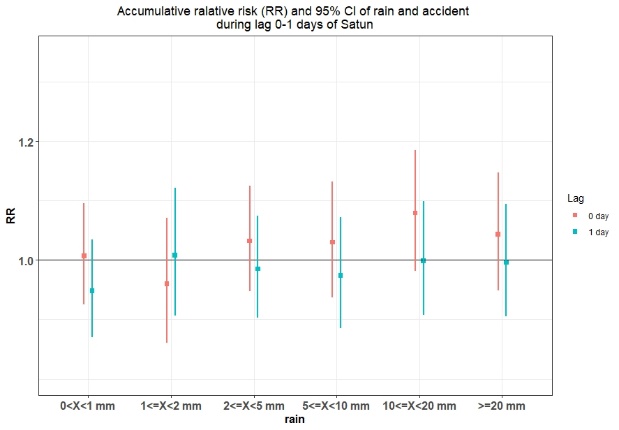 | 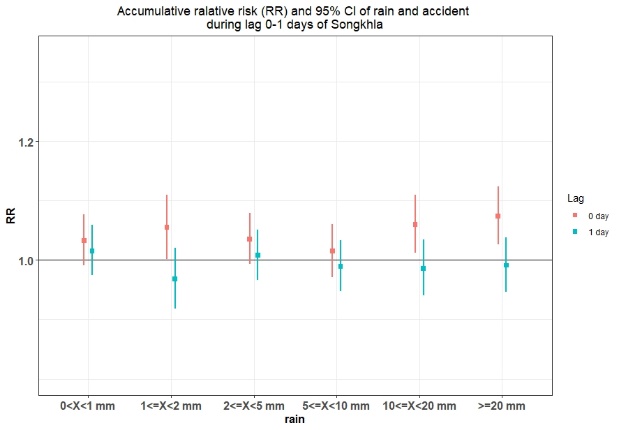 |
| Surat Thani | Yala |
| 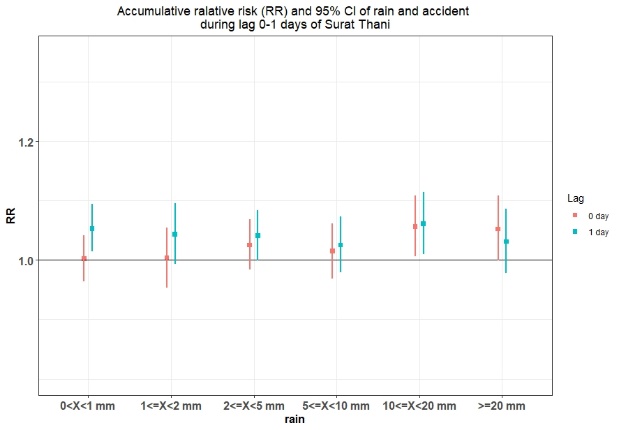 | 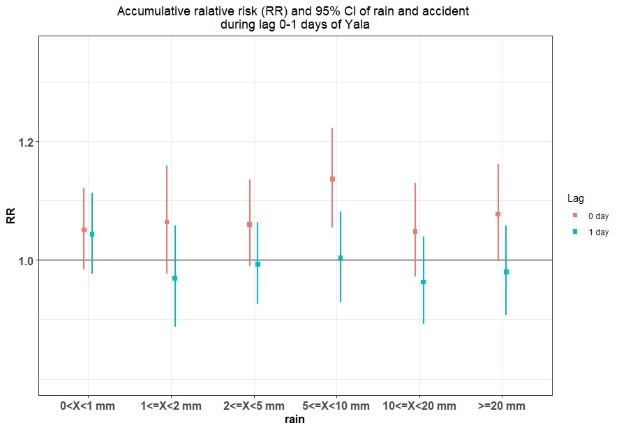 |

**Figure S2** Forest plot of the relative risk with 95% CI for road accidents with different rain group groups and different lagged days of province in the Northern and the Southern provinces. The DLNM package in the R software is allowed to study delay effects (lags) for providing the estimated effect between rainfall and road accidents via function cross-basis.

**Table S1** Summary daily statistics for temperature in Thailand during 2012-2018 over Northern and Southern provinces. All values in °C.

| **Province** | **Mean ± SD** | **Percentiles** | | | | |
| --- | --- | --- | --- | --- | --- | --- |
|  |  | **Min** | **P25** | **P50** | **P75** | **Max** |
| **Temperature (**°C**)** | | | | | | |
| **Northern provinces** | | | | | | |
| Chiang Rai | 25.3±3.1 | 8.9 | 23.4 | 26.0 | 27.4 | 33.6 |
| Chiang Mai | 27.0±2.7 | 11.5 | 25.6 | 27.4 | 28.7 | 35.1 |
| Nan | 26.7±2.9 | 9.6 | 25.3 | 27.3 | 28.6 | 33.8 |
| Payao | 25.7±3.1 | 9 | 24.0 | 26.3 | 27.8 | 34.9 |
| Phrae | 27.0±2.8 | 10.9 | 25.7 | 27.4 | 28.6 | 36.2 |
| Mae Hong Son | 26.3±3.3 | 15.3 | 24.3 | 26.7 | 28.2 | 35.6 |
| Lampang | 26.9±2.9 | 10.5 | 25.6 | 27.3 | 28.7 | 35.3 |
| Lamphun | 26.8±2.9 | 10.9 | 25.3 | 27.2 | 28.6 | 35.6 |
| Uttaradit | 28.1±2.5 | 12.1 | 26.8 | 28.3 | 29.5 | 36.6 |
| **Southern provinces** | | | | | | |
| Krabi | 27.0±1.2 | 23.4 | 26.2 | 27.0 | 27.8 | 31.2 |
| Chumphon | 27.4±1.4 | 21.2 | 26.5 | 27.5 | 28.3 | 31.9 |
| Trang | 27.6±1.3 | 23.5 | 26.8 | 27.6 | 28.4 | 31.8 |
| Nakhon Si Thammarat | 27.5±1.3 | 23.5 | 26.6 | 27.5 | 28.4 | 31.3 |
| Narathiwat | 27.5±1.2 | 22.6 | 26.8 | 27.6 | 28.4 | 31.1 |
| Pattani | 27.6±1.2 | 23.4 | 26.8 | 27.7 | 28.5 | 31.8 |
| Phang Nga | 27.7±1.2 | 23.4 | 27.0 | 27.9 | 28.6 | 31.5 |
| Phatthalung | 27.8±1.2 | 23.4 | 27.0 | 27.8 | 28.5 | 31.4 |
| Phuket | 28.7±1.2 | 24.4 | 28.0 | 28.8 | 29.6 | 32.2 |
| Ranong | 27.4±1.3 | 23.7 | 26.5 | 27.4 | 28.2 | 32.0 |
| Satun | 27.9±1.1 | 24.1 | 27.1 | 28.0 | 28.7 | 32.0 |
| Songkhla | 28.8±1.2 | 23.1 | 27.4 | 28.2 | 29.0 | 32.5 |
| Surat Thani | 27.3±1.3 | 22.6 | 26.4 | 27.2 | 28.1 | 32.9 |
| Yala | 27.3±1.3 | 23.1 | 26.5 | 27.3 | 28.1 | 32.0 |

Abbreviations: SD: standard deviation, P_x x_th: percentile, Min: minimum, Max: maximum

**Table S2** Summary daily statistics for relative humidity in Thailand during 2012-2018 over Northern and Southern provinces. All values in %.

| Province | Mean ± SD |  | Percentiles | | |  |
| --- | --- | --- | --- | --- | --- | --- |
|  |  | Min | P25 | P50 | P75 | Max |
| **Relative humidity (%)** | | | | | | |
| **Northern provinces** | | | | | | |
| Chiang Rai | 76.5±8.3 | 45.0 | 72.0 | 78.0 | 82.0 | 95.0 |
| Chiang Mai | 70.1±10.6 | 39.0 | 63.0 | 72.0 | 78.0 | 96.0 |
| Nan | 76.7±8.5 | 51.0 | 72.0 | 78.0 | 82.0 | 98.0 |
| Payao | 77.9±9.5 | 40.0 | 73.0 | 80.0 | 84.0 | 98.0 |
| Phrae | 76.0±9.3 | 43.0 | 70.0 | 78.0 | 83.0 | 97.0 |
| Mae Hong Son | 75.8±11.1 | 40.0 | 69.0 | 80.0 | 84.0 | 95.0 |
| Lampang | 74.1±10.3 | 41.0 | 68.0 | 76.0 | 81.0 | 96.0 |
| Lamphun | 73.1±11.2 | 40.0 | 66.0 | 75.0 | 81.0 | 98.0 |
| Uttaradit | 71.6±9.7 | 39.0 | 65.0 | 72.0 | 79.0 | 95.0 |
| **Southern provinces** | | | | | | |
| Krabi | 83.34±7.3 | 58.0 | 79.0 | 84.0 | 88.0 | 99.0 |
| Chumphon | 81.44±5.9 | 60.0 | 78.0 | 81.0 | 85.0 | 98.0 |
| Trang | 81.1±7.5 | 58.0 | 77.0 | 82.0 | 87.0 | 98.0 |
| Nakhon Si Thammarat | 82.82±5.8 | 59.0 | 79.0 | 83.0 | 86.0 | 100.0 |
| Narathiwat | 81.4±4.6 | 67.0 | 79.0 | 81.0 | 84.0 | 97.0 |
| Pattani | 81.0±5.3 | 59.0 | 77.0 | 81.0 | 84.0 | 98.0 |
| Phang Nga | 83.6±6.6 | 50.0 | 80.0 | 84.0 | 83.6 | 88.0 |
| Phatthalung | 82.1±5.7 | 63.0 | 78.3 | 82.0 | 86.0 | 98.0 |
| Phuket | 81.0±6.9 | 56.0 | 72.0 | 77.0 | 81.0 | 97.0 |
| Ranong | 79.9±7.8 | 56.0 | 75.0 | 81.0 | 86.0 | 95.0 |
| Satun | 79.2±7.2 | 55.0 | 75.0 | 80.0 | 84.0 | 96.0 |
| Songkhla | 77.8±5.7 | 62.0 | 74.0 | 77.0 | 81.0 | 96.0 |
| Surat Thani | 83.8±5.7 | 65.0 | 80.0 | 84.0 | 88.0 | 99.0 |
| Yala | 81.0±6.1 | 54.0 | 77.0 | 81.0 | 85.0 | 99.0 |

Abbreviations: SD: standard deviation, P_x x_th: percentile, Min: minimum, Max: maximum
